# Digital Innovation in Medicinal Product Regulatory Submission, Review, and Approvals to Create a Dynamic Regulatory Ecosystem—Are We Ready for a Revolution?

**DOI:** 10.3389/fmed.2021.660808

**Published:** 2021-05-21

**Authors:** Judith C. Macdonald, David C. Isom, Daniel D. Evans, Katy J. Page

**Affiliations:** ^1^Global Regulatory Policy and Intelligence, Pfizer, Tadworth, United Kingdom; ^2^Global Regulatory Policy and Intelligence, Pfizer, Groton, CT, United States; ^3^Global Biometrics and Data Management, Pfizer, Sandwich, United Kingdom; ^4^Global Regulatory Operations, Pfizer, Sandwich, United Kingdom

**Keywords:** regulatory, digital, dynamic, cloud, review, artificial intelligence (AI), machine based learning, ecosystem

## Abstract

The pace of scientific progress over the past several decades within the biological, drug development, and the digital realm has been remarkable. The’omics revolution has enabled a better understanding of the biological basis of disease, unlocking the possibility of new products such as gene and cell therapies which offer novel patient centric solutions. Innovative approaches to clinical trial designs promise greater efficiency, and in recent years, scientific collaborations, and consortia have been developing novel approaches to leverage new sources of evidence such as real-world data, patient experience data, and biomarker data. Alongside this there have been great strides in digital innovation. Cloud computing has become mainstream and the internet of things and blockchain technology have become a reality. These examples of transformation stand in sharp contrast to the current inefficient approach for regulatory submission, review, and approval of medicinal products. This process has not fundamentally changed since the beginning of medicine regulation in the late 1960s. Fortunately, progressive initiatives are emerging that will enrich and streamline regulatory decision making and deliver patient centric therapies, if they are successful in transforming the current transactional construct and harnessing scientific and technological advances. Such a radical transformation will not be simple for both regulatory authorities and company sponsors, nor will progress be linear. We examine the shortcomings of the current system with its entrenched and variable business processes, offer examples of progress as catalysts for change, and make the case for a new cloud based model. To optimize navigation toward this reality we identify implications and regulatory design questions which must be addressed. We conclude that a new model is possible and is slowly emerging through cumulative change initiatives that question, challenge, and redesign best practices, roles, and responsibilities, and that this must be combined with adaptation of behaviors and acquisition of new skills.

## Introduction

We live in a time of transformation and accelerated change. Rapid advancement in our understanding of the biological basis of diseases, genomic science, informatics, and digital health over the past several decades is yielding breakthrough therapies that change patient’s lives (1). This is fueled both by novel approaches to generate evidence using new sources of information (such as real-world data, patient experience data, and digital biomarkers), and by a drive toward patient centric development. Meanwhile, clinical trial conduct is being transformed, for example by using decentralized approaches that leverage remote monitoring and reduce the burden on patients traveling to visit clinical sites. All these innovations are enabled by digital technology—this generation’s “steam engine” and what has been referred to as the 4th industrial revolution (2).

Innovation in the submission, review and approval of regulatory data on medicinal products has also progressed over the last few decades, primarily focused on standardization of formats and efficiency of operations. However, without a radical re-imagining of this approach, it will not be possible to fully embrace broader advances in science and digital technology. Regulatory authority review and approval of medicinal products still largely relies on construction and exchange of electronic versions of paper documents. Thus, valuable data are locked away in formats that impede update or re-use resulting in regulatory processes with discrete and often unconnected milestones for interaction. Further, bespoke and convoluted workflow processes still differ across both regulatory authorities and company sponsors and are so entrenched and hardwired that they will be challenging to de-construct. This transactional model of static and intermittent exchanges between regulatory authorities and company sponsors obstructs a holistic and iterative view of data supporting a medicinal product’s efficacy, safety, and quality profile related to its intended use.

In short, a radical digital transformation of the approach for regulatory submissions and review is needed to allow for dynamic contemporaneous updating of regulatory data. The Covid-19 pandemic has highlighted the need for rapid secure exchange between regulatory authorities to understand the basis of decisions and accelerate global approvals. This will require a fresh look at the multiple industry and regulatory authority digital platforms. A secure shared solution could facilitate valuable collaboration between regulatory authorities to maximize their resources and enhance efficient regulatory authority reviews and approvals across the globe. Such a re-imagined model would ultimately be able to accommodate the global use of new evidence sources such as real world evidence from electronic health records, wearable health devices, and exploit digital tools such as machine-based learning (MBL), and artificial intelligence (AI). Regulatory authority decision making would be enriched through access to new evidence sources, such as non-applicant generated external data, and broader product context through identification of common trends across similar products.

**Figure 1:**
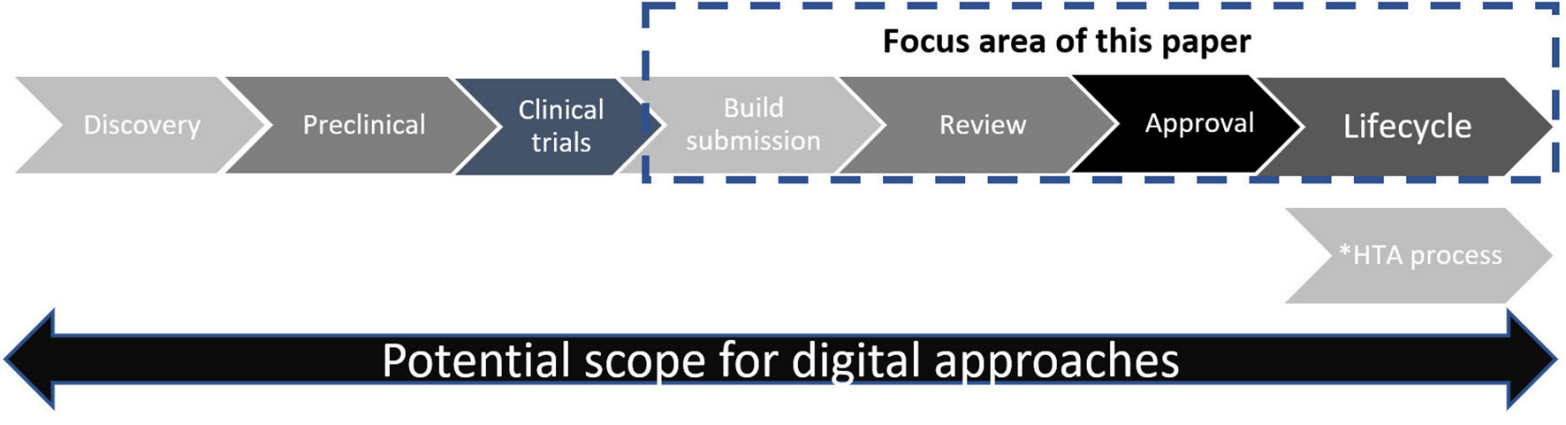
Simplified schematic of drug development and review. ^*^Timing of HTA process varies according to national procedures—in some countries HTA review may start in parallel to regulatory view.

In this article, we share our perspectives on current challenges in regulatory submission and review procedures (aka “pain points”) and identify regulatory design questions that help us navigate toward a new model. While potential benefits span the entire research, development and lifecycle spectrum (Figure 1), we focus solely on opportunities to transform interactions between company sponsors and regulators via the late-stage processes of submission build, review, and approval [i.e., issue of a Marketing Authorization (MA)] and lifecycle management.

An ultimate aspiration would be a secure regulatory ecosystem that accommodates all stakeholders who use information beyond company sponsors and regulators [e.g., health care professionals, Health Technology Assessors (HTAs), patient advocacy groups, individual patients, and academics]. With necessary safeguards and controls, this ecosystem could enable a learning healthcare system^1^. A roadmap for this is beyond the scope of this article. However, we also believe that as each iterative innovation (use case) is progressed with this aspiration in mind, a roadmap is slowly emerging.

## Current MA Submission and Review Process: Limitations and Challenges

Thanks to the work of the International Congress for Harmonization (ICH) in standardizing content, structure, and format of regulatory submissions via the Common Technical Document (CTD) and the electronic CTD (3–6) company sponsors and regulators have benefited from and have longstanding familiarity with largely consistent expectations, notwithstanding locally required content. Online navigational aids (hypertext links) have created the ability to move across hundreds or thousands of individual documents, and company sponsors can submit and update their marketing authorization applications (MAAs) to multiple agencies without having to re-format the majority of the information. Importantly, multidisciplinary review and information sharing became much easier.

With standardization the focus, formal exchanges between company sponsors and regulators have been predicated on sequential and prescriptive document transactions in the form of regulatory submissions. Introduction of electronic submissions and eCTD digitalized these files (Adobe acrobat Portable Document Formats (PDFs)^2^ but the basic system of review and exchange of product information between company sponsors and regulators remains a static “snapshot in time” via individual documents, with changed content requiring new or replaced versions. Content remains trapped within documents hampering the application of new digital tools. The persistent burden of content update across individual interdependent documents is significant and open to error. Similarly, the cumbersome nature of access and extraction of PDF content, where needed by regulators for internal review templates and for companies to re-use, neatly illustrates the inadequacy of this outdated document based model. Additionally, use of external real world evidence in submissions is increasing. Information and data needs demand that we unlock the full potential of machine based learning and artificial intelligence to successfully interrogate and integrate diverse sources of evidence—company-generated clinical studies and real world data—for the benefit of current and future patients.

### Clinical Data Challenges

The focus of the current transactional model on data sequestered mainly within Adobe PDF documents produces significant inefficiencies and challenges for clinical documentation. These include maintaining the critical linkage between the protocols, statistical analysis plans, study results within clinical study reports and subsequent responses to regulatory queries containing additional or amended results. The lack of linkage or synchronization between the design process (regulatory scientific advice, protocol review, statistical analysis plans, and programing specifications), study outcome (study report text and tables), and the subsequent regulatory review and queries leads to significant inefficiencies and delays to decision making. Advanced analytical tools such as semantic search and visualization make possible the linkage of all these materials, offering the potential to transform regulatory interactions during these stages while delivering substantial process efficiencies. The use of additional tools relying on the data and algorithm standards, offer the potential to perform rapid analyses of the clinical datasets supplied to the FDA, for example, in order to verify and explore the outcomes from clinical trials whilst maintaining the linkages between all this information. These solutions have the potential to deliver a more effective platform which will enhance the review of all submission information related to clinical trials.

### Chemistry Manufacturing and Control (CMC) Data Challenges

Submissions for regulatory review of CMC/Quality follow complex bespoke company processes to aggregate information from a variety of sources (including methods and assays from laboratory and manufacturing equipment) to build tabulations and develop different summaries for multiple countries. CMC content populates relevant sections of the CTD and is generally in CTD/eCTD format that is globally harmonized at a high level. Beyond the high level harmonization, there are variations in information and ancillary documents by country. After receipt, regulators may then manually extract the text-based information from the tables in PDF forms via copying or re-transcribing to import the data into their internal databases, workflow, and evaluation tools.

As with Clinical and Safety, CMC information is continually updated from early research through to MA and beyond via post marketing changes, where it can be even more complex. Across the globe there is typically a delay in regulator review and approval of post approval changes (often CMC), resulting in a queue of changes awaiting action/review by national regulators due to lack of risk-based approaches (especially in emerging markets), forced sequencing, and/or limitations in regulatory authority review resources. This issue is identified as contributing to medicine shortages (7). Therefore, there is considerable advantage in a future ability to release real time updates simultaneously to multiple regulators post approval.

### Envisioning a New Model for Regulatory Submissions and Review

There is a need to transform the submission, review, and exchange of data between company sponsors and regulators in approval of medicinal products. A cloud based platform (or equivalent) could house a much more dynamic and iterative exchange. For example there could be a series of data rooms, an individual company sponsor only data room where data could be uploaded in a continuous fashion as each submission component is finalized, a shared room between the company sponsor and the regulatory authority where they may interact on review issues and a regulatory authority—only room where the regulator will conduct confidential review and will interact internally with reviewers in the same health authority. Such an approach could enable a more dynamic and iterative exchange between regulatory authorities and company sponsors unlocking some time efficiencies and creating more of a “living system” which houses all current data supporting the product. Adobe acrobat PDFs could be broken up and data structured in databases rather than documents—allowing more efficient abstraction and analysis. The benefits of this approach could continue post approval and facilitate CMC post approval change management by reducing bottlenecks via a more contemporaneous update and exchange. This approach could also provide a secure platform for regulator to regulator collaboration for example in a work-sharing or reliance setting. Over time further efficiencies could be unlocked by the application of machine based learning and artificial intelligence as data would be in formats more amenable to this. Benefits could include automation of routine tasks to save resources and identification of trends in data via digital tools. This would also allow greater use of modeling and simulation which could unlock new insights. Such a model would be more amenable to incorporating data from non-traditional sources such as real world data.

### Progress to Date

Substantial industry investment is being made to advance this new model. Accumulus Synergy, established as a not-for-profit standalone organization in 2020 with initial funding from several leading pharmaceutical companies^3^, represents a significant step forward (8). The long-term vision is to transform the dialog between regulatory authorities and company sponsors by defining the future of data exchange, clearly aligning with the model envisaged in this article. Accumulus-Synergy is actively working with regulatory authorities such as the US FDA, the EMA, the Pharmaceutical and Medical Devices Agency (PMDA) in Japan and others to define the path forward through practical means—for example, by establishing initial use cases that benefit both industry and regulators by tackling common pain points. This is a new approach which is not merely optimizing current document based transactional systems but is re-imagining an entirely new approach. Core capabilities will be developed incrementally with the aim of these producing scalable global solutions. There is recognition of the need to prove value by building short term capability and yet not lose sight of the long-term vision which will radically transform regulatory submissions, review and approvals, ultimately enabling efficiencies via artificial intelligence and machine-based learning.

Furthermore, several regulatory authorities are already pursuing digital modernization strategies to enhance their IT capacity and data management, and advance analytics capabilities to keep pace with the rapidly evolving scientific and technology aspects of digital Research and Development (R&D). Recent examples are the FDA Technology and Data Modernization Action Plan (TMAP) (9), and the European medicines regulatory network telematics strategy (10). In addition to these modernization plans and strategies, regulatory authorities are also engaged in numerous standards organization based data initiatives (e.g., ISO IDMP and HL7’s Vulcan), Public Private Partnerships (e.g., IMI initiatives), industry collaborations (Transcelerate), submission, and review data standardization and knowledge initiatives (e.g., FDA’s PQ/CMC and KASA) (Table 1).

**Table 1:** Examples of platforms/initiatives advancing data standardization, knowledge application, data sharing, and utilizing new forms of evidence.

**Platform/initiative summary**	**Advancing the Healthcare sector to a new Paradigm through:**
Pharmaceutical Quality/Chemistry manufacturing and Controls, (PQ/CMC, A HL7^1^ sponsored US FDA data standardization initiative pursuing standardized [eCTD Module 3] CMC data and format, moving away from the PDF based requirement, to a structured data model (11). It also includes streamlined population of assessment templates by reviewers and leveraging of workflow management tools relevant to inspections, and a proof of concept to assess the feasibility of HL7 as a data exchange solution for PQ/CMC sponsored by the HL7 Biomedical Research and Regulation (BR&R) Work Group^2^	Informed decision making through structured data exchange between FDA and sponsors, enhancing understanding of context and precedence through use of internal tools for structured review, automated workflow, and knowledge management such as KASA
International Organization for Standardization (ISO), comprising national standards bodies in 165 countries, has developed *Identification of Medicinal Products (IDMP)* as a controlled standard vocabulary. IDMP is referenced by FDA and EMA in their data standardization efforts but largely in use in Europe. Standardized terms will facilitate inter-operability of systems (12)	Common and unique identifiers for pharmaceutical products and substance information through data standardization. Applications in pharmacovigilance, clinical trials, regulatory submissions, and GMP inspections (12)
Vulcan, launched by Health Level Seven^®^ International (HL7^®^), seeks to use its widely recognized data exchange standards to facilitate collaboration among diverse stakeholders in the translational and clinical research community to define a common set of standards that can be implemented internationally (13)	Effective acquisition, exchange, and use of data in translational and clinical research using data exchange standards to promote interoperability across healthcare and clinical development
Knowledge Aided Assessment and Structured Application program (KASA), used by US FDA in Generic drugs, mining data to recognize patterns and trends across different applications. Potential for broader FDA adoption with added risk assessment support (14)	Enhanced internal workflow and learning through knowledge sharing across applications and efficiency of review through data mining
TransCelerate is a not-for-profit biopharmaceutical organization that has pioneered improvements in clinical research and development, specifically collaboration and data sharing. Examples include Common clinical trial protocol template; and DataCelerate^®^ a global cloud-based data sharing platform that allows for deidentified, anonymized pre-clinical, and clinical data types to be requested and voluntarily shared in a secure and data compliant way (15)	Reusable content, “cloud” collaboration and data sharing through structured reusable content to streamline clinical development data operations
Innovative Medicines Initiative (IMI), offers two examples of projects focusing on novel healthcare evidence sources such as EHDEN for electronic health records (European Health Data and Evidence Network) (16) and BD4BO, (Big Data for Better Outcomes) (17)	Secure Data network using common data models for healthcare data to inform clinical practice, and promote clinical research

1 *https://confluence.hl7.org/display/HE/HL7+Essentials*.

2 *https://confluence.hl7.org/display/BRR/Pharmaceutical+Quality+%28PQ%29+PSS*.

Some regulatory authorities are advancing initiatives to also address the CMC data management challenge. The FDA Pharmaceutical Quality and CMC (PQCMC) effort (Table 1), for example, seeks to standardize data and format elements of the CMC submission in eCTD Module 3, moving away from the PDF based requirement to a structured data one (11). This is expected to bring several advantages: decrease reviewer time and effort to populate assessment templates and tools, leverage workflow management tools relevant to inspections, inform decision making with enhanced understanding of context and precedent, and optimize workflow using tools such as the Knowledge Aided Assessment and Structured Application (KASA) program used by FDA for generic drugs, (Table 1). ICH is also considering improvements to CTD quality documentation, endorsing a revision to the M4Q guidance on 27 May 2020 (18).

## Regulatory Implications for a New Model for Regulatory Submissions and Review

**Figure 2:**
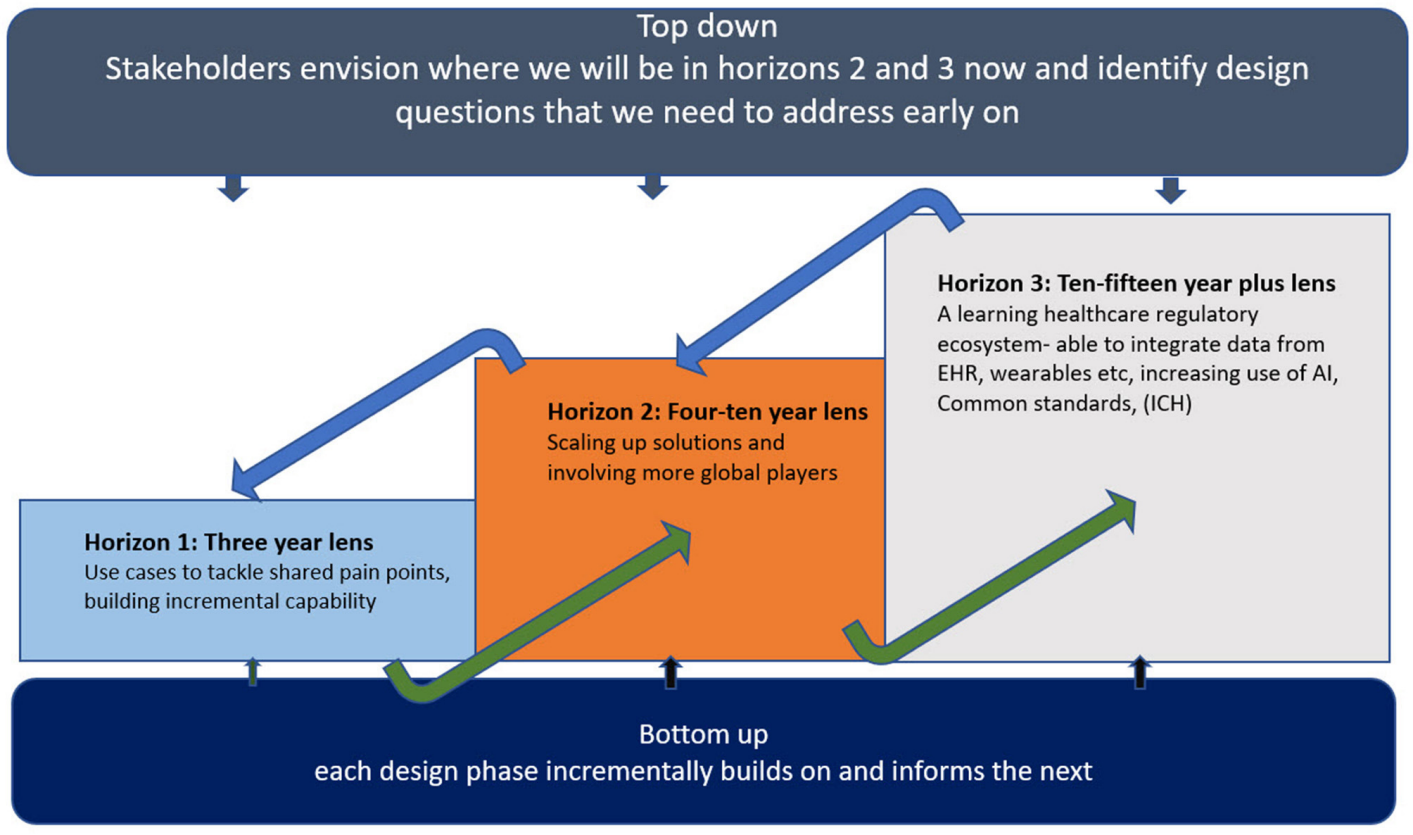
Three horizons for transformative change.

The new model outlined here will take time and require a balanced approach to accommodate all stakeholder viewpoints. Successful execution will depend on both building value through incremental use of practical use cases in the short term (e.g., via Accumulus -Synergy’s current activities) while maintaining the longer-term ambition (Figure 2).

**Figure 3:**
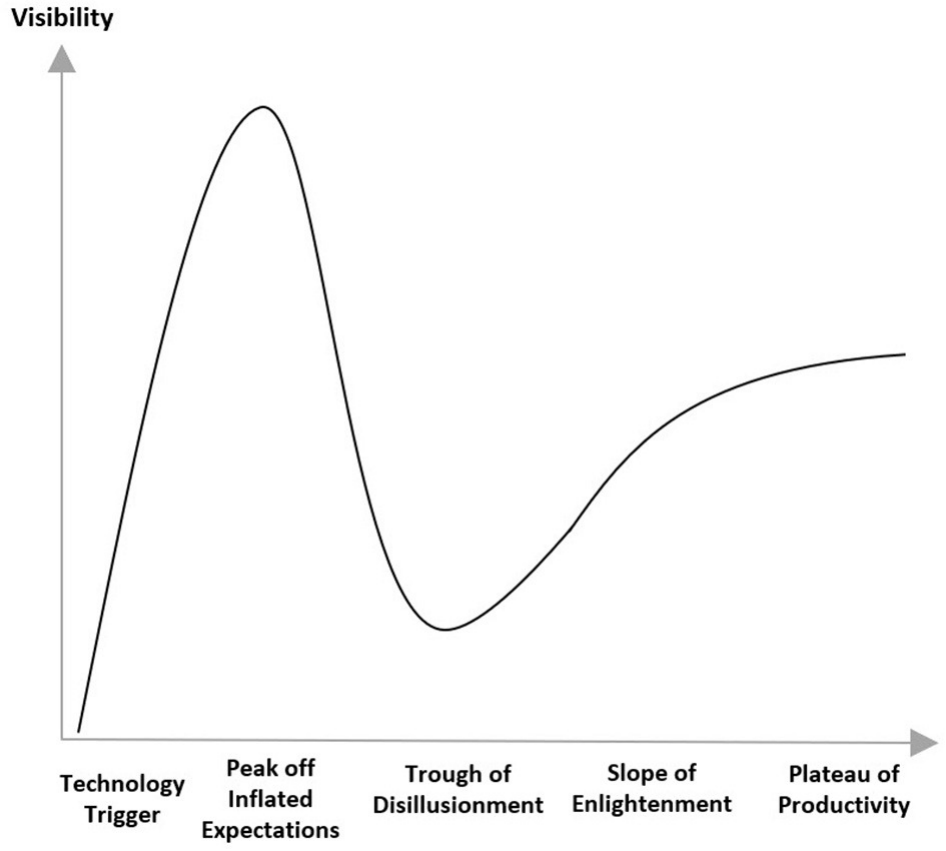
Hype cycle (20).

It’s important not to underestimate the challenges. Fenn and Blosch (19) illustrate the typical course of introduction of new technology as five phases. Initially an innovation trigger leads to a peak of inflated expectations followed by a trough of disillusionment. Ultimately as realistic expectations of capability emerge, there is a slope of enlightenment followed by a plateau of productivity (Figure 3).

It is important to realize that this new model is not simply moving the existing approach to submissions into a new storage repository (cloud), rather it will require a comprehensive process and requirements re-design. Submission build will be more iterative and incremental with the possibility of a closer to real time exchange of data with the regulatory authorities, without being restricted to milestone based meeting dates. Below we identify some design questions and implications in moving toward the new model. While a detailed analysis of the solutions to these questions is beyond the scope and intention of this article, we hope to initiate and encourage multi-stakeholder dialog. Ultimately, we aim to contribute to both calming the “hype curve” by avoiding overly optimistic extremes, and collectively attaining the slope of enlightenment more quickly.

### To What Extent Will There Be a Move From a Document Driven to a Data Driven Approach?

As mentioned earlier, Adobe PDF documents comprise much of the information in regulatory submissions. This format is not optimal to exploit artificial intelligence and machine-based learning tools. Initiatives are underway to develop structured content management systems whereby a database holds human and machine-readable blocks of information and allows importation of such data into linked documents (21). Any change to such documentation can be made once only and automatically applies to all linked content. This database approach holds considerable advantage in improving both data integrity and re-usability. However, we suggest that there is still critical value added via company sponsor authored summaries. One example being the clinical overview which brings a strategic and expert clinical opinion to positioning of data messages, and cross functional linkages to preclinical and CMC content such as justifying clinically relevant specifications. Therefore, we do not envisage that all submission components will move from a document driven to a data driven approach, at least in the short term.

### What Is a Regulatory Submission and How Is It Defined?

Today, submission of an application to the regulatory authorities is developed over time and formally submitted by the company sponsor once the last document is available. Each submission must contain all the data and information required for review and, if digital, is typically published with navigational aids before digital upload to the regulator. The upload then requires regulatory authority acknowledgments, in most cases, both initial receipt, followed by confirmation that construction and format requirements have been met through successful processing via the regulatory authority portal and/or technical validation tool. These control steps take place prior to content review.

The new paradigm takes this digital upload approach further. Data and information may theoretically be uploaded by the company sponsor into a secure virtual environment “cloud” as each defined “data packet” is ready, at that point becoming available for regulatory authorities to access the required data or information on a product by pulling down from the cloud to effectively start to assemble the submission according to their regulatory requirements and potentially start review for each individual component without waiting for all components to be uploaded. Another option would be for company sponsors to upload data as each component is finalized, but only open regulatory authority access once all components are ready for review. Digital upload and access, e.g., the definition of a “data packet,” will require precise rules and standards. Different design rules may need to apply or be desirable depending on the type of review and the product.

The FDA OCE’s pilot Real Time Oncology Review (RTOR), operates in the current regulatory framework while allowing earlier provision of clinical data shortly after all patient data has been entered and locked by the applicant in their database. Earlier FDA feedback on clinical data has resulted in company provision of additional analyses and proven benefit in reduced approval timelines (22). Although this is not actually a true real time continuous upload and iterative review process, it is a significant innovation that moves us a step closer to this new model. However, there are resource implications and process adjustments arising from a more iterative approach both for company sponsors and regulatory authorities since it potentially requires new rules on when and how queries would be triggered/expected and timelines for responses. Initially these approaches may be limited to products with high unmet need. Unlocking further efficiencies via enhanced use of AI and MBL is likely needed before more widespread application could be considered.

### What Is a Marketing Authorization Approval and What Is It Based on?

In the current paradigm, there is the concept of pre-approval before the regulatory authority has reached a decision to approve the product, and post approval whereby the product can be made available commercially. After (“post”) approval, companies must continue to submit safety, efficacy, and manufacturing updates to ensure the product holds contemporary information throughout its life cycle, and where licensed for use. There are different levels of product knowledge, expectations, and obligations before and after approval, so this distinction is important.

Data uploaded in real time into a cloud-based data sharing environment, as mentioned above, potentially blurs the distinction between pre and post approval data flow. Company sponsors generally need predictability of timing for approvals to plan manufacture of launch supplies. A need remains for the regulatory authority to make a point in time decision on whether sufficient evidence has been submitted to grant the MA and make it available to patients. In this scenario, as with the aforementioned point on incremental data release, it will be critically important to secure a clear mutual agreement between regulatory authorities and company sponsors on when and how the threshold and criteria for initial approval(s) have been met and the expected projected timeframe for the final decision given the potential for continuous and contemporaneous updating of information. It will also be important for company sponsors and regulatory authorities to preserve a freeze frame record of the knowledge base at the time of approval as without this the initial basis for the approval will be without context and unclear.

There is further potential to enrich regulatory authority decision making with digital tools expected to make it easier to refer to external data sources/algorithm analyses and/or identify trends applicable across similar classes of medicines. Learnings across products can be facilitated by developing new constructs on re-use/pooling of data to enable a broader context to be built around the review. In the future, Regulatory authority decisions for a single product may no longer be based predominantly on the data sources submitted by the individual company sponsor. External data/analyses may be leveraged and be confidential to the regulatory authorities and use could be reasonably expected to increase over time as AI and MBL tools become more widely applied. Without line of sight into external analyses performed, it could be challenging for the company to understand the basis of decisions or understand the basis of queries received from regulatory authorities in assessment. On the other hand, company sponsors may also seek to access these data sources to enrich their submissions with external data/analyses via digital tools. Regulatory authorities would then need to assess the rigor and validity of such data/analyses alongside more classically generated sponsor research. The MA would need to record the external data and analytics used. Also, careful thought will need to be given to the inclusion of such external data, whether generated by the MA Holder or not, in the approved label for the product and hence how this may impact company sponsor promotional activities.

### Will This New Model Enable Universal Dossier Content to Be Submitted Simultaneously to All Global Regulatory Authorities and a Single Regulatory Review Decision in a Similar Timeframe?

The ICH aim to achieve a standardized two-way global submission and approval process has been accepted by many as the gold standard. We fully recognize the significant achievements made, however equally significant are the real and ongoing challenges with contradictory and complex national transactional approaches. In our view, an ultimate aspiration of one single dossier worldwide, enabled by a common technical document (CTD and eCTD) or future cloud based system, does not stand up to scrutiny beyond the obvious and compelling case for logistical efficiency and speed. On closer examination, this focus on an optimally efficient single output and virtual environment ignores critical factors that drive decisions on national approval and access to medicines. Such factors include varying public health needs and priorities particularly with respect to interpretation of clinical data^4^ and how the product is expected to be used relative to any existing therapies (e.g., first line use, second line use, etc.). These factors can drive divergent regulatory authority approval decisions and/or different labeling recommendations even when core data are standardized.

Despite progress by ICH, not all regulatory requirements are harmonized and not all countries are ICH members; national and regional requirements still exist in addition to common core information. In other words, submitting the same core information in 40 markets may be initially efficient and appear superficially attractive, but would not result in 40 approvals at the same time due to differing review times/requirements and those approvals would not all look the same. Once lifecycle work is then initiated the single output then multiplies and diverges further due to complexities with post approval change management, though the aforementioned ability in the future state to file contemporaneous post approval updates to multiple markets may well offset this as will implementation of ICH Q12.

Aside from differing regulatory requirements and approval timelines mentioned above, other constraints exist in considering filing a universal MAA to numerous countries simultaneously. For example, it is possible that manufacturing capacity limitations may still constrain the ability of a pharmaceutical sponsor to supply multiple markets at the same time even if there were to be approvals within a similar timeframe. Another consideration would be the capacity for company sponsors to handle the increased volume in multiple regulatory authorities’ queries coming in within a compressed time window.

Further, before patients can access therapies in many markets, national HTA approval is also required. These national reviews often occur after regulatory review and can become rate limiting for patient access once regulatory authority reviews are shortened and optimized. Our message here is that aligned regulatory submissions will not result in aligned approvals or patient access.

For all the above reasons, we believe that it is too simplistic to expect that the new model will automatically enable a “one size fits all” approach to the entire regulatory submission content with a single global review and approval. However, we note that there could be better prospects for the possibility of a more universal content approach for the CMC module rather than the clinical modules, and though still beset with differences in laws and business processes, and interpretations of data between national regulatory authorities, this may be more attainable in the long term (23). However, we contend that the overarching goal should not solely be operational efficiency but rather, patient-centric regulatory authority decisions, based on redefined approaches to review and approval of contemporaneous product safety, efficacy and quality data, underpinned by available digital capabilities. In moving toward this goal, all stakeholders should seek to converge national regulatory requirements as far as possible toward a universal approach, but it is not by itself the ultimate goal, nor is a cloud-based system the sole enabler.

There is an emerging trend for regulatory authorities with similar capabilities and philosophies to engage in collaborative work sharing or reliance approaches^5^. Such approaches necessitate a high level of trust and a degree of commonality of review approaches. A good current example of work-sharing is the ACCESS consortium. This is a collaborative initiative of like-minded, medium-sized regulatory authorities between Australia’s Therapeutic Goods Administration (TGA), Health Canada (HC), Singapore’s Health Sciences Authority (HSA), the Swiss Agency for Therapeutic Products (Swissmedic) and more recently the Medicines and Healthcare products Regulatory Authority (MHRA) of the United Kingdom (24). Participating regulatory authorities allocate responsibilities for review of different CTD modules. Though ultimately the approval decisions remain a national responsibility, collaboration enhances the efficiency of review. For legal reasons the US FDA cannot participate in worksharing via the ACCESS consortium, however, Project Orbis is an initiative from FDA’s Oncology Center of Excellence to enhance global collaboration and review specifically for oncology products. Orbis engages a similar set of national regulatory authorities to ACCESS, but in this case they benefit from FDA’s review (as opposed to work-sharing), which allows their national decisions to be expedited (25).

Not all regulators are resourced to conduct even a partial review of submissions and may elect instead to recognize the approval conducted by a larger health authority based on the provision of a Certificate of Pharmaceutical Product or CPP instead of conducting their own review. However, increasingly, as the WHO seeks to strengthen regulatory systems such that all regulators achieve basic capability (26), this type of pure recognition without any form of review is not as widespread as in the past with many health authorities conducting their own review in addition to receiving a CPP (27).

Overall therefore, we envisage the future state could realistically consist of several formalized regional networks of regulatory authorities with similar review approaches, working together via collaborative work-sharing or reliance reviews based on shared review practices and similar public health needs. The new cloud based model could enhance and accelerate this type of collaboration.

### What Are the Regulatory Operations Considerations?

Persistent divergence in national regulatory data and format requirements across developed and developing markets for obtaining and maintaining a MA (content, language, construction, and format), and the resulting complexity and multitude of outputs required has led to certain operational capabilities addressing these divergences being matured as a core competence. Regulatory authority and company sponsor efficiencies have largely centered on refining transactions, automating sub processes or functions, adjusting capacity and prioritization. Many have invested in incremental improvements and independent technology solutions, while company sponsors aspire to an integrated process, data, and digitalization strategy across all stakeholders.

To date, we suggest that company sponsors have primarily focused on developing their internal data and information management skills and strategy with regulatory authorities out of scope. Successful transformation will surely require expansion and investment to accommodate both the co-existence of current transactional models and transition to a new model inclusive of company sponsor and regulatory authority needs (28). As stated earlier, moving to a “cloud” in of itself does not fundamentally alter the landscape though a useful catalyst for change. Companies investing in change initiatives with regulatory authorities, (examples described in Table 1), must be realistic in accepting more cost, divergence, work and risk pursuing an MA approval within horizon 1 (Figure 2) before reaping the benefits attained at horizons 2 and 3. This operational challenge may be more acute for large multinational companies needing to manage the span of different approaches and speeds of adoption across multiple national regulators. Learnings from CTD and eCTD inform us that the path to a new model will not be quick nor linear, however, with digital technology available the pace of change could feasibly accelerate once initial test cases demonstrate benefit.

### What Changes in Review Practices/Upskilling and Behavioral Adaptations May Be Needed?

The overarching driver is to provide safe and effective medicines through optimal assessment of all available data and information. Both a critical element and challenge, is the needed evolution in human behaviors (29). Company sponsors and regulatory authorities must consider their talent management strategy, investing in workforce skills for the future by training their regulatory scientists to be digitally literate as well as scientifically strong. Adjusting the assessment paradigm will need changes in information management and review skills and practices. The ability to access and analyze data/algorithms from other sources to enrich product knowledge and inform regulatory decision making will be a critical expertise, as will task efficiency through automation of more routine aspects, again supported by digital tools. Such a resource intensive effort would surely require further investment in scientific and technical skills, therefore targeted elimination of manual effort will also likely be necessary for advancement.

### What Are the Global Considerations?

Consideration needs to be given to how an acceptable standard of regulation around the world could be accelerated by this digitally enabled model (30). We believe that driving toward more use of reliance and work sharing procedures between regulatory authorities will need to go hand in hand with pioneering a new model, since it is becoming increasingly clear that even the most well-resourced regulators do not have the capacity to be entirely self-sufficient (31). Secure platforms that facilitate exchange of information between regulators, and provide transparency on the review approach, will enhance trust and encourage the use of reliance to deliver further efficiencies in getting products approved in multiple geographies, particularly if regulatory networks and work-sharing arrangements increase as previously suggested.

It may be inevitable and appropriate that a paradigm shift of this nature will initially need to be driven, tested and pioneered by well-resourced company sponsors and regulatory agencies. However, this should not imply that its design should ignore the needs of company sponsors and/or regulatory authorities with fewer resources. It should be possible to consider this perspective from the start by keeping in mind how practical a proposal could be when rolled out more widely and what level of IT infrastructure and funding would ensure that these economies are not left behind. Implementation of CTD/eCTD is resource intensive. Smaller regulatory authorities with fewer resources may decide not to invest in CTD implementation and wait for the cloud based model instead. In our view, the ultimate vision is going to take many years to perfect and there is value in applying CTD approaches in the interim as an incremental step forward. It is equally possible that regulators with simpler processes could have an advantage over others that have invested in CTD/eCTD as they have less complexity in current business processes to dismantle. Political and socioecomic factors will continue to be a significant influence on progression.

### What Considerations Are Needed on Data Access Rules, Data Quality, Security, Confidentiality, and Intellectual Property?

Principles around use and safeguarding of data (e.g., blinded and unblinded), ownership, and interdependencies [e.g., definition of interim safety and efficacy analyses and supportive documentation (e.g., protocols)], will need comprehensive exploration to clarify needed controls, decision making and approval processes.

Together with data and information standards and rules for use, this is one of the most important considerations which requires extensive discussion beyond the scope of this article. Suffice to say that data access rules and data transfer procedures will need to accommodate stringent rules for patient data privacy in each country. From both a company sponsor and a regulatory authority perspective it will be vital to have strong safeguards in place to guarantee security of information such that confidential proprietary information does not inadvertently enter the public domain. A move to a new model will demand rigorous systems to ensure patient data privacy and safeguard intellectual property while allowing secure data collaboration.

In the longer term, it is envisaged that the model can be expanded to benefit broader stakeholders such as HTAs, academics and patients. Enhanced accessibility of data may increase third party (academia or national HTA) *post-hoc* analyses of clinical data that reach a different conclusion to the regulatory authorities. This will require meticulous consideration of how to avoid data analyses or conclusions being taken out of context, to avoid inadvertent undermining of decisions and erosion of public confidence and trust.

Data quality and integrity is another critical aspect. Over many years, the industry has perfected systems to assure the quality and integrity of the data it generates, and this is checked by regulators via inspections for Good Clinical Practice (GCP), Good Manufacturing Practice (GMP), and Good Pharmacovigilance Practice (GVP). Such safeguards must continue, and we will need to look for ways to apply similar quality standards to other data sources.

### What Learnings Can We Glean From Past Initiatives/Other Industries?

The pharmaceutical industry has partnered with trade associations, regulators, and researchers to demonstrate how shared third-party computing environments can enable novel ways to exchange regulatory information in support of clinical research and regulatory review. Major initiatives include CRIX/FIREBIRD (Clinical Research Information Exchange/Federal Investigator Registry of Biomedical Information Research Data) (32), OMOP (Observational Medical Outcomes Partnership) (33), and ASTER (ADE Spontaneous Triggered Electronic Reporting) (34).

Each of these successfully demonstrated new ways to leverage available technology to both establish a shared industry and regulator platform and manage data and information. All demonstrated potential to innovate traditional processes. CRIX FIREBIRD demonstrated a collaborative platform for credentialing new investigators for clinical trials. OMOP established a collaborative scientific platform to establish standards and methodologies for evaluating associations between drugs and health outcomes, and ASTER demonstrated a third-party service for automatically processing individual adverse event reporting directly from electronic health records to FDA and sponsors. All had significant industry and regulator engagement. Although these novel data exchange initiatives were not ultimately adopted to replace traditional processes as envisioned, learnings from these efforts informed initiatives such as Transcelerate and FDA’s Sentinel (medical product safety surveillance).

Cloud-based technology services are widely available today and remove some of the previous challenges addressed through investment in expensive, customized technology platforms, like those noted above. However, other more fundamental barriers faced in those initiatives remain. These previous endeavors have taught us that the biggest struggle in implementation relates to the complexity of business processes between regulatory authorities and company sponsors. These have evolved over time as tightly woven webs around the current paradigm. Unraveling such complexity will involve defining new roles, governance, processes, principles, and data strategies that are accepted globally in lieu of current norms. At the same time, there will be a need to ensure global investments in technology modernization to ensure all regulators have the minimum computing infrastructures needed. None of this investment can interrupt the pursuit of new medicines to patients.

Of note, though not explored here, other highly regulated sectors such as finance have successfully disrupted their business model though innovative use of technology and undoubtedly there are learnings to be gained worthy of further exploration (35).

## Actionable Recommendations

We offer the following recommendations:

- Through trade associations and regulator networks, continue to debate, refine and expand on the regulatory implications noted earlier to deliver patient centric solutions.
- Pursue harmonization of all types of regulatory data requirements including but not limited to terminology and structured content management initiatives and press for these to be harmonized and inter-operable between countries.
- Through Accumulus Synergy cloud-based pilots, assess small scale use cases in collaboration with regulators to build value, considering global implications from the outset and scaling up capabilities in terms of ambition and scope over time.

## Discussion

Digital innovation can propel stakeholders to radically redesign the burdensome and time consuming processes involved in review and approval of new medicines. This is not simply taking the existing eCTD approach and putting it into a cloud-based platform but re-imagining and re-designing the entire process for interaction between regulators and company sponsors.

In this article, we have made the case for change for regulatory submissions and review by showing that the current approach is not fit for the future and by outlining a cloud based model to transform submission and review to be more iterative, collaborative and dynamic. We have touched on a few, but by no means all, of the regulatory implications of this new approach. Each of the regulatory design questions posed could easily be explored as publication in its own right and there are further discussion points that we have not explored (e.g., data ownership). All questions that we start to socialize here need further input and refinement from all stakeholders. We would expect that new questions will arise as organizations such as Accumulus-synergy execute use cases. Use cases also yield valuable learnings which can inform and sharpen the focus for a long term vision for the future where ultimately other stakeholders such as HTAs, academia, and patient groups can also benefit from this approach. We contend that the extent to which we are able to identify, socialize, and further debate the implications of change indicates the extent of our readiness to embrace the revolution needed to create a dynamic regulatory exchange and review system, fit for the future that fully leverages all available science and technology. This new approach does not remove the need to pursue the harmonization of regulatory requirements via ICH so that drug development is science based. Continued pursuit of convergence and harmonization initiatives will avoid any inadvertent risk that unrestricted storage capacity in a cloud system encourages regulatory creep whereby non-science based country or regional requirements proliferate. Regulatory reliance and work-sharing initiatives are a key consideration and expansion of these initiatives should also be pursued as regulatory authority resources are constrained. A cloud based system provides the perfect platform to expand and increase the efficiency of these type of initiatives.

However, there is also a cost in pursuing this new model. At the operational level company sponsors must invest in its design while also maintaining the existing approach in parallel for some years as not all countries will be able to embrace this change immediately. Accumulus-synergy is an important vehicle as it allows company sponsors and regulatory authorities to work toward the new model, separate from the need to keep the usual cycle of submissions thus reviews on track using the current system. New iterative data upload and review has resource implications for company sponsors and for regulatory authorities thus will likely require a phased approach in the interim period before efficiencies are unlocked via use of digital tools. Both company sponsors and regulatory authorities need to invest in upskilling their workforces for a more digitally based future. There needs to be a willingness to dismantle business processes which have evolved over many years fixated on a paper based mind-set. A cloud based platform encourages collaboration and should make data sharing easier and more secure. It could be considered that the Covid-19 pandemic has changed some of the contours of the regulatory landscape including clinical data sharing expectations. There have been calls for company sponsors to commit to a new interpretation of what is regarded as competitive data and share more than has been done in the past (36). Indeed, if company sponsors wish to access publicly held data sources such as real world data via electronic health records they may be called upon to reciprocally release more of their in-house data whilst also upholding intellectual property considerations. This and other questions require further discussion.

Despite the cost of pursuit and the reality of maintaining the existing approach whilst rolling out the new model incrementally across countries, the benefits unlocked by this new approach far outweigh the expenditure in effort. These benefits include the possibility to make regulatory submissions and reviews more efficient by enabling a more contemporaneous exchange of data and facilitating parallel reviews between regulators. Breaking free from the constraints of PDF documents enables use of digital tools which can also reveal new insights into data facilitate data reuse in related submissions and automation of more routine tasks. Life-cycle management can be made much more efficient removing the forced sequencing of changes awaiting individual national regulatory authority review. Facilitating use of non-traditional data sources such as real world evidence or data from wearable health devices alongside traditional clinical trial data is ultimately expected to enrich regulatory decision making and benefit patients. There is a long way to go, however, we are encouraged that industry and regulatory authorities can be prepared to embrace this revolution by continuing to socialize and debate the considerations outlined in this article. We contend that the benefits of pursuing this approach are tangible and attainable. Are we ready to embrace the full benefits of this 4th revolution? We cannot afford not to be.

## Author Contributions

All authors were involved in developing the concept and methodology as well as drafting and editing the manuscript. All authors reviewed the final version.

## Conflict of Interest

All authors are employees and shareholders in the pharmaceutical company Pfizer.

## Footnotes

^1^A Learning Healthcare System is defined, by the Institute of Medicine (IoM) (Institute of Medicine 2015), as a system in which, “science, informatics, incentives, and culture are aligned for continuous improvement and innovation, with best practices seamlessly embedded in the delivery process and new knowledge captured as an integral by-product of the delivery experience.”

^2^A minority of regulators (e.g., US FDA) also require raw data sets and programs but for most regulatory authorities the submission comprises of PDF components.

^3^At the time of writing members included Amgen Astellas, Bristol Myers Squibb, Eli Lilly, GlaxoSmithKline, Johnson and Johnson, Pfizer, Roche, Sanofi, and Takeda.

^4^There can also be differences in interpretation of CMC data that impact labeling and occasionally overall approvability if there are significant regulatory concerns. Usually, however, clinical data interpretation differences are the main driver for divergent decisions.

^5^WHO Technical Report Series 1025, Annex 10, (March 2021), Good Reliance Practices defines **Reliance** as “*the act whereby the NRA in one jurisdiction may take into account and give significant weight to assessments performed by another NRA or trusted institution, or to any other authoritative information in reaching its own decision. The relying authority remains independent, responsible, and accountable regarding the decisions taken, even when it relies on the decisions and information of others.”* **Work-sharing** is defined as “*a process by which NRAs of two or more jurisdictions share activities to accomplish a specific regulatory task.”*
